# Response to “Investigating procedural safety: comparative analysis of rotational atherectomy and modified balloon angioplasty” by Tang et al.

**DOI:** 10.1007/s00392-024-02559-3

**Published:** 2024-10-23

**Authors:** Alexander Maier, Mark Colin Gissler, Constantin von zur Mühlen

**Affiliations:** 1 Department of Cardiology and Angiology, University Heart Center Freiburg—Bad Krozingen, Freiburg, Germany; 2 Faculty of Medicine, University of Freiburg, Freiburg, Germany

Sirs,

We appreciate the interest shown by Tang et al. in our article, “Procedural safety of rotational atherectomy and modified balloon angioplasty: insights from a German national registry” [1] and express our appreciation for their efforts in commenting on numerous research articles across various medical disciplines and specialties in recent months. The following responds to the points raised.

First, patients with target vessel thrombosis, hemodynamic instability, extreme vessel tortuosity and angulation, pre-dilation dissection, vein graft lesions, or coronary artery bypass grafts were not excluded from our study. The dataset used from DeStatis does not fully capture all mentioned clinical characteristics. The percentage of patients with previous cardiac surgery is reported in Table 1. As stated in our manuscript, we excluded patients with acute coronary syndrome.

Second, our study did not report on diagnosed hyperlipidemia and previous (multivessel) PCI. However, our analysis accounted for severity of underlying coronary artery disease by propensity score methods and inverse probability weighting for presence of coronary 1, 2, 3 and/or left main disease as well as previous myocardial infarction.

Finally, the safety of crossover strategies between MB and RA, or vice versa, was not explicitly investigated in our study. Initially, our analysis included patients with and without crossover treatment strategies. The results are reported in our article. In an additional analysis, excluding the 1179 patients who were treated with both MB and RA, the results remained consistent, which is also reported in the article. The outcomes of patients treated with intentional or unintentional crossover strategies need to be investigated in future studies.

We thank Tang et al. for their discussion and comments on our article. Further research is warranted to fully elucidate the points raised.
